# On strata damage and stress disturbance induced by coal mining based on physical similarity simulation experiments

**DOI:** 10.1038/s41598-023-42148-4

**Published:** 2023-09-19

**Authors:** Yi Yang, Yingchun Li, Lujun Wang, Yang Wu

**Affiliations:** 1 School of Energy and Mining Engineering, China University of Mining and Technology-Beijing, Beijing, 100083 China; 2 State Key Laboratory of Water Resource Protection and Utilization in Coal Mining, National Institute of Clean and Low Carbon Energy, Beijing, 102211 China; 3 Deep Underground Engineering Research Center, Dalian University of Technology, Dalian, 116024 China

**Keywords:** Energy science and technology, Engineering

## Abstract

Extensive studies have been conducted on the movement of overlying strata when a single coal seam is mined. However, structural characteristics and associated stress field variation of the overlying strata over multiple coal seam mining remain unclear. Here we performed physical modelling experiments analogous to No. 42108 working face of Buertai coal mine, Shendong coalfield, where No. 22 coal seam (2.9 m thickness) was mined first, preceding No. 42 upper coal seam (6.1 m thickness) with an inter-coal-seam distance of 72.8 m. We employed DIC (digital image correlation) measurement and systematically-laid pressure cells to visualize the overlying strata movement and monitor stress field variations over multiple coal seam mining. We found that the stress of the inter-coal-seam strata increased significantly in the late mining stage of No. 22 coal seam due to the strata collapse, and culminated after compaction of the caved blocks. The inter-coal-seam strata stress gradually decreased over mining of No. 42 upper coal seam and arrived at zero after the inter-coal-seam strata collapsed. The mining of No. 42 upper coal seam aggravated the roof settlement of No. 22 coal seam; and the floor stress was noticeably lower than that of No. 22 coal seam due to the pressure-relief caused by the former mining activity. Our physical modelling findings advanced our understanding on structural characteristics and stress evolutions of overlying strata over multiple coal seam mining and offered guidance for prediction and mitigation of strata movement associated disasters in underground coal mining with geomechanical and mining conditions similar to those of Buertai coal mine.

## Introduction

China coal production in 2022 reached 4.5 billion tons, occupying 56.2% of the national energy consumption. More than 80% of the coal production is contributed from underground coal mining. As the coal field resources in the middle and east of China is depleting, the coal production proportion of northwestern coal mines grows significantly, among which Shendong coalfield is the most prominent. The underground coal mining in Shendong coalfield typifies high-intensity (annual production over 30 million tons for a single coal mine), relatively shallow burial depth (150–450 m), large mining height (up to 8.8 m) and multiple coal seam mining^1–3^, which commensurably lead to remarkably different overlying strata movement and structural characteristics from previous studies^4–6^.

When a coal seam is mined underground, the overlying strata experience deformation, breakage, collapse and caving into the goaf^7^. Due to the movement of the overlying strata, three zones are typically developed above the working face of the coal seam, namely, caved zone, fractured zone and bending zone^8^. The characteristics of the overlying strata movement from the coal seam roof to the surface heavily depend on geomechanical properties of the strata^9,10^ and mining conditions including mining depth^1,5,11^, working face geometry^12^, advancement speed^13^, mining height^2,14^, mining method^15^, the number of coal seams and the distance between these coal seams under extraction^16–19^. Understanding the strata movement and associated stress evolution is closely related with ground pressure occurrence^2,6,20^, underground water flow^21,22^, gas drainage^23,24^ and mining-induced surface subsidence^4,11,15^. Different approaches have been employed to examine field-scale features of strata movement, including theoretical analysis^25,26^, numerical modelling^9,10,27–31^ and site investigations^32–34^. However, these studies commonly involved complex geological and geomechanical conditions, which made it difficult to fully illuminate the underlying mechanism since the strata were hardly visible^16^. On the other hand, down-scale physical modelling in the laboratory have been extensively leveraged to disclose the development of cracks/fractures and the overlying strata deformation/movement over the advancement of the working face^12,35–38^, mainly due to the advantages of visualization and effective analogy. Most studies of physical modelling experiments have been conducted emphasizing the overlying strata movement during the mining of a single coal seam^11,12^. Due to superimposed excavation effect and repeated stress disturbance^39–41^, the strata movement and stress field change under the mining of multiple coal seam at different levels can be much more complicated than those of a single coal seam mining, which requires systematic investigation.

Here, we took No. 42108 working face of Buertai coal mine, Shendong coalfield, China as the engineering site for physical modelling. Two coal seams were sequentially mined with No. 22 coal seam (2.9 m thickness) mined first, preceding No. 42 upper coal seam (6.1 m thickness). DIC (digital image correlation) technique and systematically arranged pressure cells were employed to reveal the overlying strata movement and stress field variations over multiple coal seam mining.

## Physical modelling experiment

### Coal mine introduction

**Figure 1:**
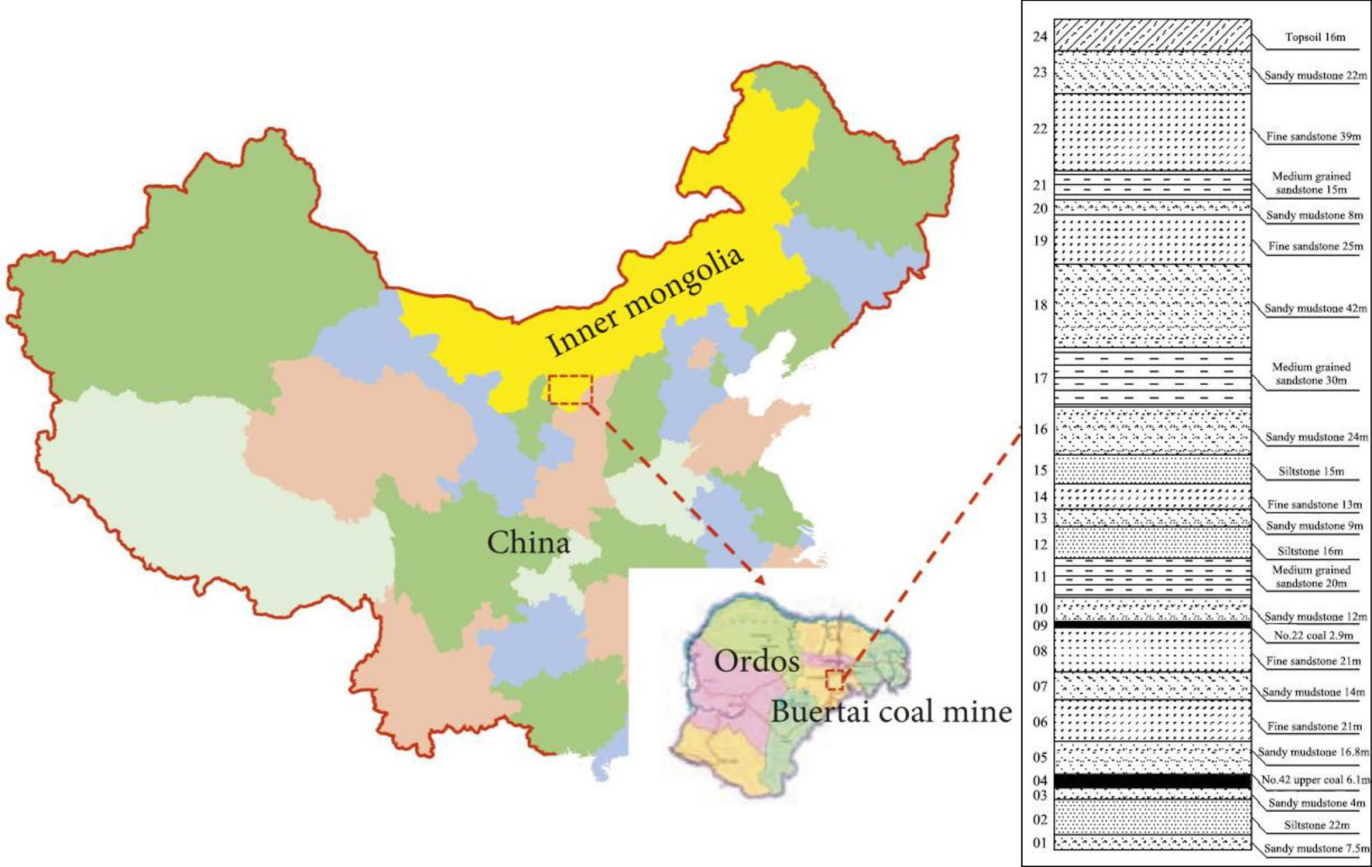
Location of Buertai coal mine and strata histogram of No. 22 coal seam and No. 42 upper coal seam.

Buertai coal mine with an annual production of 20 million tons is located in Ordos, Shendong coalfield, China (Fig. 1). The coal mine has multiple coal seams of large burial depth (390–430 m) and complex geological conditions. The coal mine mainly extracts No. 22 coal seam (average thickness 2.85 m) and No. 42 upper coal seam (average thickness 4.57 m). The average distance between the two coal seams was 72.8 m. The floor elevations of the mine field and No. 42 upper coal seam were 1150–1340 m and 880–960 m; and the maximum burial depth of the working face of No. 42 upper coal seam was about 430 m.

The longwall mining technique is used in Buertai coal mine. To study the overlying strata movement and stress evolution under multiple coal seam mining, we selected No. 42108 working face of Buertai coal mine where No. 22 coal seam (2.9 m thickness) and No. 42 coal seam (6.1 m thickness) were successively extracted. The width (dip direction) and length (strike direction) of No. 42108 working face were 313 m and 4728.4 m. The fully mechanized caving mining method was employed to excavate the coal seam and the main roof was fully caved after mining.

**Figure 2:**
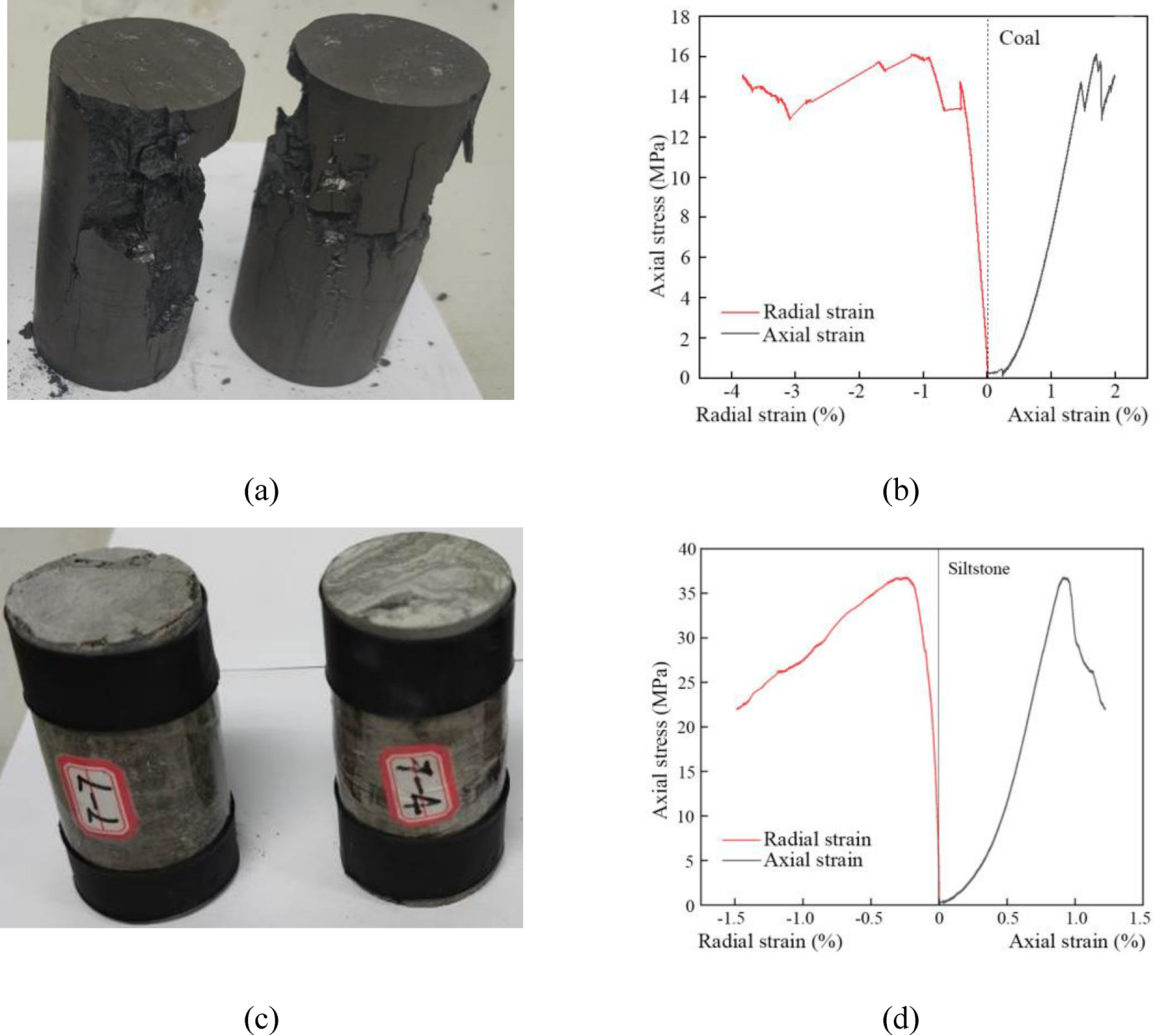
Uniaxial compression experiments. (**a**) deformed coal sample; (**b**) stress–strain curves of coal sample; (**c**) deformed siltstone sample; and (**d**) stress–strain curves of siltstone sample.

To obtain the basic physical and mechanical properties of the coal seams and overlying strata (Fig. 1), rock and coal samples acquired by in-situ coring were tested by standard uniaxial compression, triaxial deformation and indirect tension tests to determine the uniaxial compressive strength, internal friction angle, cohesion and tensile strength of these rock and coal samples (Fig. 2). These rock and coal samples can be classified into five lithologies according to their properties shown in Table 1, including medium-grained sandstone, siltstone, fine sandstone, sandy mudstone and coal.

**Table 1:** Mechanical properties of rock strata.

No.	Coal/rock	Elastic modulus (GPa)	Poisson’s ratio	Internal friction angle (°)	Cohesion (MPa)	Uniaxial compressive strength (MPa)	Tensile strength (MPa)	Uniaxial strength of analogous materials (MPa)
1	Medium-grained sandstone	5.5	0.35	33	6.1	25	1.6	0.17
2	Siltstone	7.1	0.4	37	8.8	41	3.5	0.27
3	Fine sandstone	6.1	0.14	17	11.6	35	2.5	0.23
4	No. 22 coal seam	1.7	0.5	19	14.1	21	1.5	0.11
5	Sandy mudstone	8.9	0.21	26	15.8	55	3.9	0.37
6	No. 42 upper coal seam	1.7	0.5	19	14.1	21	1.5	0.11

### Physical modelling platform

**Figure 3:**
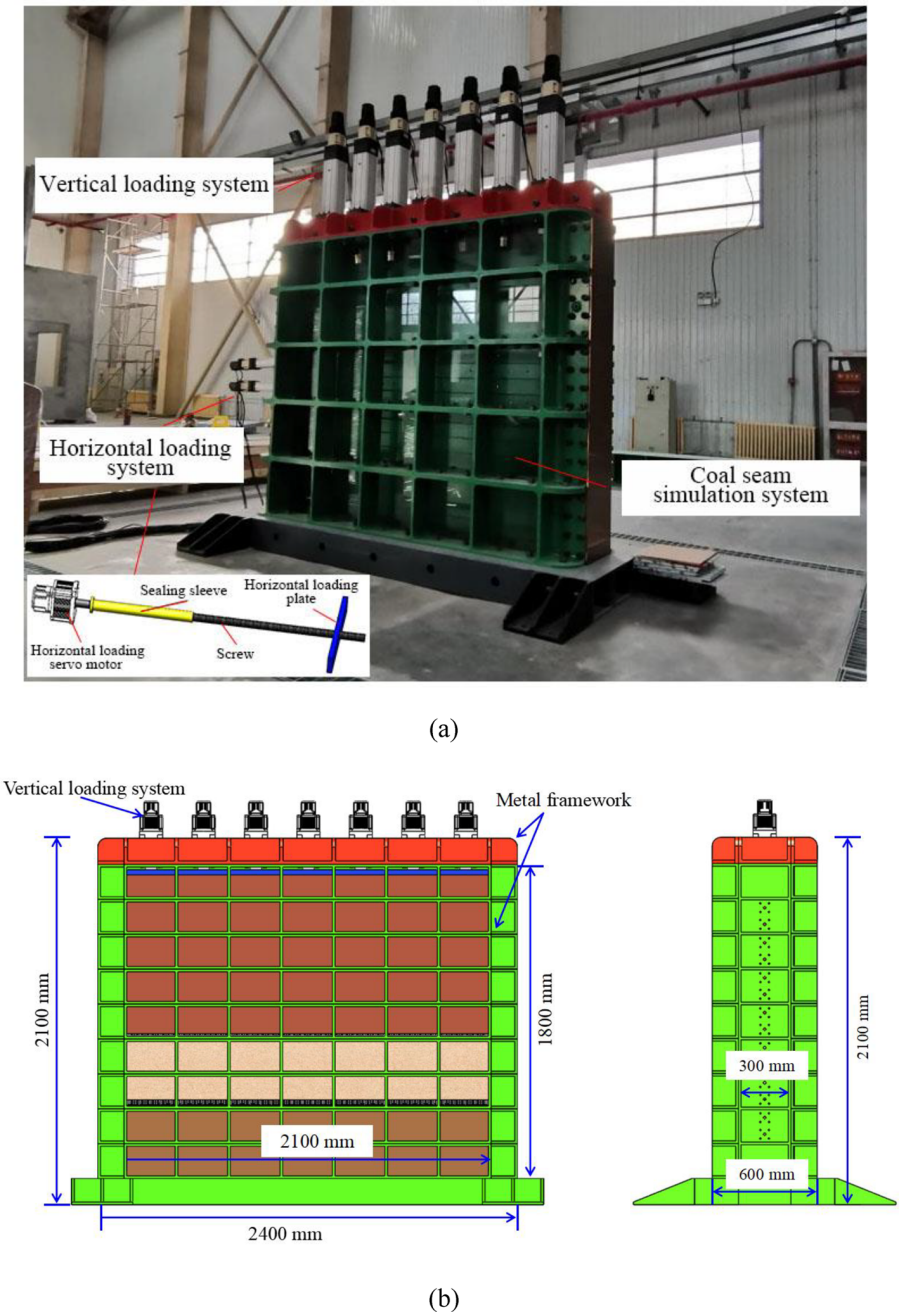
Physical modelling experimental platform. (**a**) front view; and (**b**) side view.

**Figure 4:**
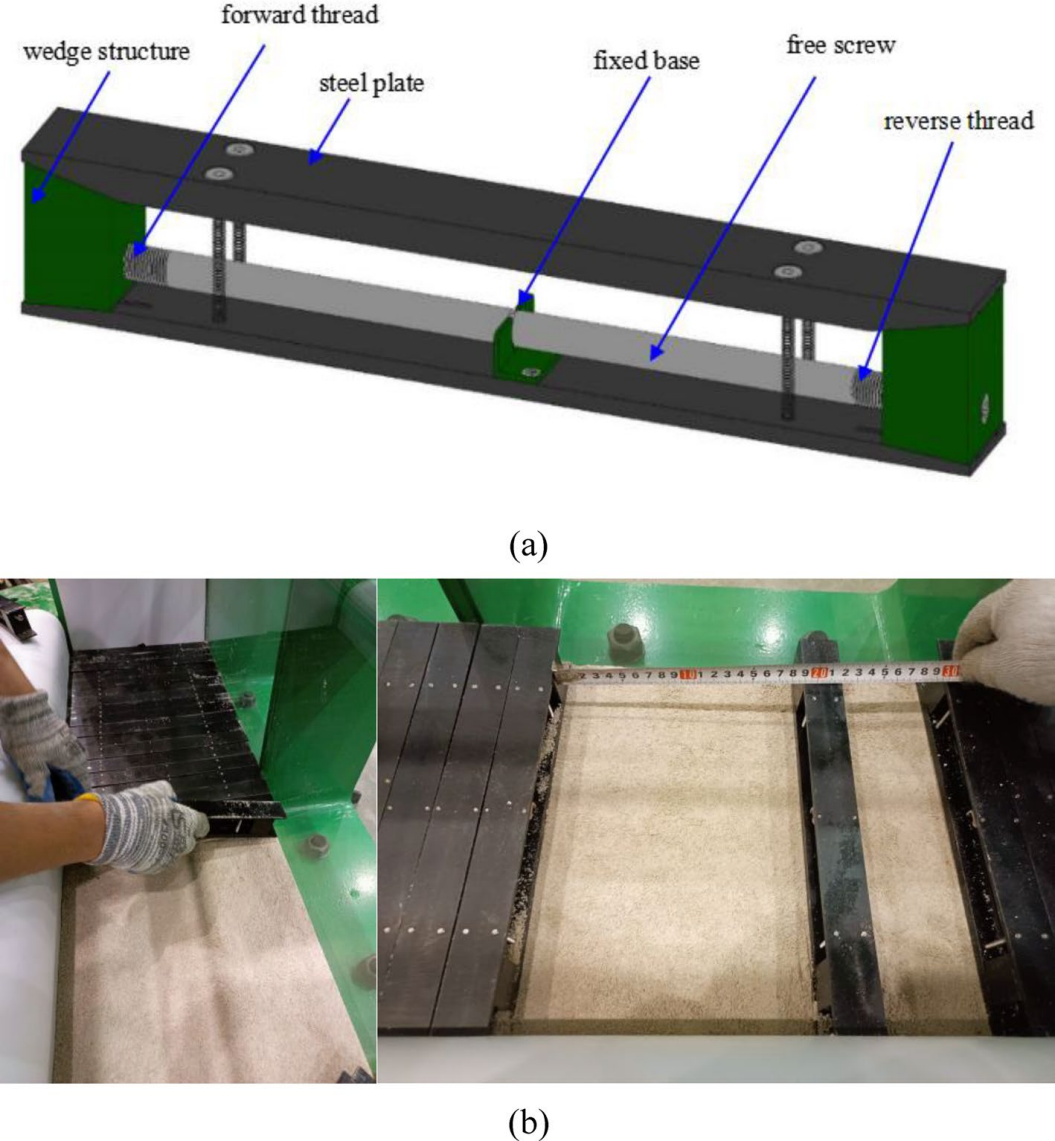
The excavation simulation device and the simulation effect of coal seam. (**a**) the excavation simulation device; (**b**) the simulation effect of coal seam.

Physical modelling experiments have been widely utilized to study strata movement over coal mining to represent field-scale scenarios^12,35–38^. In this study, we conducted the physical modelling experiment on the physical modelling experimental platform specified for studying underground coal mining with multiple coal seams developed by the State Key Laboratory of Water Resources Protection and Utilization in Coal Mining. This experimental platform can simulate the whole construction and operation processes of underground coal mining. The experimental platform mainly consisted of the main reaction frame, vertical loading system, horizontal loading system, coal seam excavation simulation device, and monitoring and acquisition system. Figure 3 demonstrates the overview of the experimental platform, including:

1. Main reaction framework
  As shown in Fig. 3, the external three-dimensional size of this whole model was 2400 mm × 2100 mm × 600 mm, and the internal size was 2100 mm × 1800 mm × 300 mm. The reaction frame was a horizontal piece-type assembly structure, and each piece was 200 mm.
2. Vertical loading system
  The vertical loading system was mainly composed of servo electric cylinder, loading plate and sealing device. It was controlled by the loading control system, which can realize step-by-step loading and continuous operation. The system height was less than 30 cm and the loading stroke was 150 mm. The stress control accuracy was ± 0.01 MPa, the displacement control accuracy was ± 0.1 mm, and the loading stress was adjustable within 0–0.5 MPa.
3. Horizontal loading system
  To apply horizontal load to the internal coal pillars, a loading hole with a diameter of 15 mm was set every 100 mm in the range of 1400 mm in the middle of the side reaction frame. Bolt holes and horizontal loading motors were set. The motor rotation drove the horizontal loading plate to move forward (Fig. 3).
4. Coal seam excavation simulation device
  The wedge structure splicing was used to simulate the expected mining portion of coal seams, and the remaining portion was composed of analogous materials. The size of this excavation device is 30 mm × 40 mm × 300 mm(width × height × length).In this experiment, the coal seam is composed of about 70 such excavation devices.The experiment simulates the gradual mining of coal seam by removing this excavation devices one by one (Fig. 4).
5. Monitoring and acquisition system
  The monitoring system was equipped with high-resolution single lens reflex (SLR) cameras, strain gauges, film stress sensors, displacement sensors and accompanied software for monitoring and acquisition system. The monitoring and data acquisition system can obtain high-resolution images and videos of the target surface. The monitored displacement, stress and strain data can be real-time displayed, stored and exported.

### Physical modelling experiment procedures

#### Physical modelling parameters

**Figure 5:**
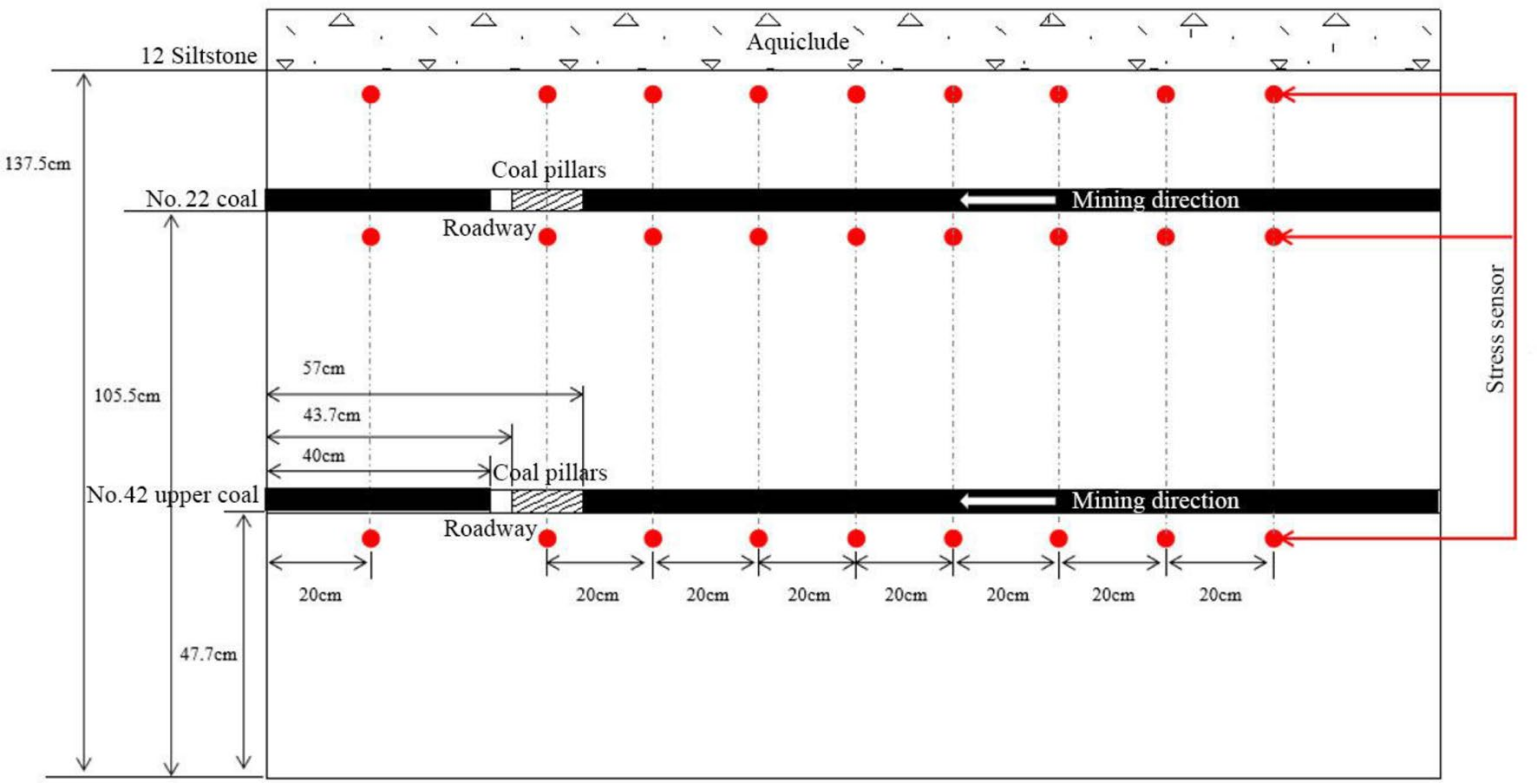
Schematic diagram of physical modelling experiment.

This physical modelling experiment took No. 42108 working face of Buertai coal mine as the engineering site, where No. 22 coal and No. 42 upper coal seams were mined successively. Pre-excavated roadways and coal pillars were set in these two coal seams, which simulated roadway development before working face advancement (Fig. 5). Considering the geometry of the experimental platform and the layout of No. 42108 working face, we simulated an advancement distance of 270 m according to a geometric ratio of 1:150. Thus, similarity ratios of the physical model utilized in this study are:

- Geometric similarity coefficient *C*_*l*_ = 1/150;
- Time similarity coefficient *C*_*t*_ = $\sqrt{{C}_{l}}$  = 1 h and 50 min;
- Bulk density similarity coefficient $C_{\gamma } = \gamma_{mi} /\gamma_{pi} = { 1}/{1}$;
- Elastic modulus similarity coefficient $C_{E} = C_{l} /C_{\gamma } = \, 1/150$;
- Strength similarity coefficient $C_{\sigma c} = C_{l} /C_{\gamma } = \, 1/150$;
- Poisson’s ratio similarity coefficient $C_{\mu } = \mu_{mi} /\mu_{pi} = \, 1$.

#### Physical model preparation

This experiment simulated the movement of the overlying strata over multiple coal seam mining. To replicate the overlying strata, cement was used as a binder, barite powder, talcum powder, silicone oil and quartz sand were used as fillers, and mica powder were laid between layers for separation. Strata of different properties were obtained by changing the ratio of the analogous ingredients. The mechanical parameters of the real strata are shown in Table 1, and the ingredients to make analogous materials of the strata in the physical model are shown in Table 2.

**Table 2:** Analogous materials to model rock strata in the physical modelling experiment.

No.	Name	Laying thickness (cm)	Total thickness (cm)	Model quality (kg)	Quartz (kg)	Baryte (kg)	Talc powder (kg)	Cement (kg)	Silicon oil (kg)	Water (kg)
16	Sandy mudstone	17.8	180	247	134.9	38.3	66.7	7.1	4.9	24.7
15	Medium sandstone	10	162.2	139	78.5	21.5	37.5	1.5	2.8	13.9
14	Fine-sandstone	8.7	152.2	121	67.4	18.8	32.7	2.1	2.4	12.1
13	Sandy mudstone	6	143.5	83	45.3	12.9	22.4	2.4	1.7	8.3
12	Siltstone	10.7	137.5	148	81.7	22.9	40	3.4	3	14.8
11	Medium grained sandstone	13.3	126.8	184	104	28.5	49.7	1.8	3.7	18.4
10	Sandy mudstone	8	113.5	111	60.6	17.2	30	3.2	2.2	11.1
9	No. 22 coal seam	4	105.5	55	31.4	8.5	14.9	0.2	1.1	5.5
8	Fine-sandstone	15.3	101.5	212	118	32.9	57.2	3.9	4.2	21.2
7	Sandy mudstone	9.3	86.2	129	70.4	20	34.8	3.5	2.6	12.9
6	Fine-sandstone	14	76.9	194	108.1	30.1	52.4	3.4	3.9	19.4
5	Sandy mudstone	11.2	62.9	155	84.6	24	41.9	4.5	3.1	15.5
4	No. 42 upper coal seam	4.0	51.7	55	31.4	8.5	14.9	0.2	1.1	5.5
3	Sandy mudstone	27.7	47.7	384	209.7	59.5	103.7	11.1	7.7	38.4
2	Siltstone	14.7	20	204	112.6	31.6	55.2	4.6	4.1	20.4
1	Sandy mudstone	5.3	5.3	73	40	11.3	19.7	2	1.5	7.3

The physical model preparation procedures are:

1. The required ingredients for casting analogous materials (barite powder, talcum powder, cement, silicone oil) were prepared.
2. The casting plates were put on both sides of the platform to set the simulated rock strata, and its height increased with that of the model.
3. The ingredients were weighed according to the prescribed proportioning, followed by adequate mixing and stirring with water.
4. According to the rock strata histogram of Buertai coal mine, the mixture is transferred to the platform, evenly laid, and then flattened with a flat plate. A level gauge was used to ensure the rock strata were flat and horizontal.
5. To ensure the physical model mimic the real strata movement, a layer of mica powder was evenly distributed on the surface of the previous layer to prevent the adhesion of adjacent two layers.
6. Thick coal seams were created by superimposing repeated layers. The appropriate thickness of each layer was 1.0–2.0 cm, and the thickness of some layers was 2.0–3.0 cm, which were proportional to the thickness of the real strata histogram of Buertai coal mine.

### Physical modelling experiment procedures

**Figure 6:**
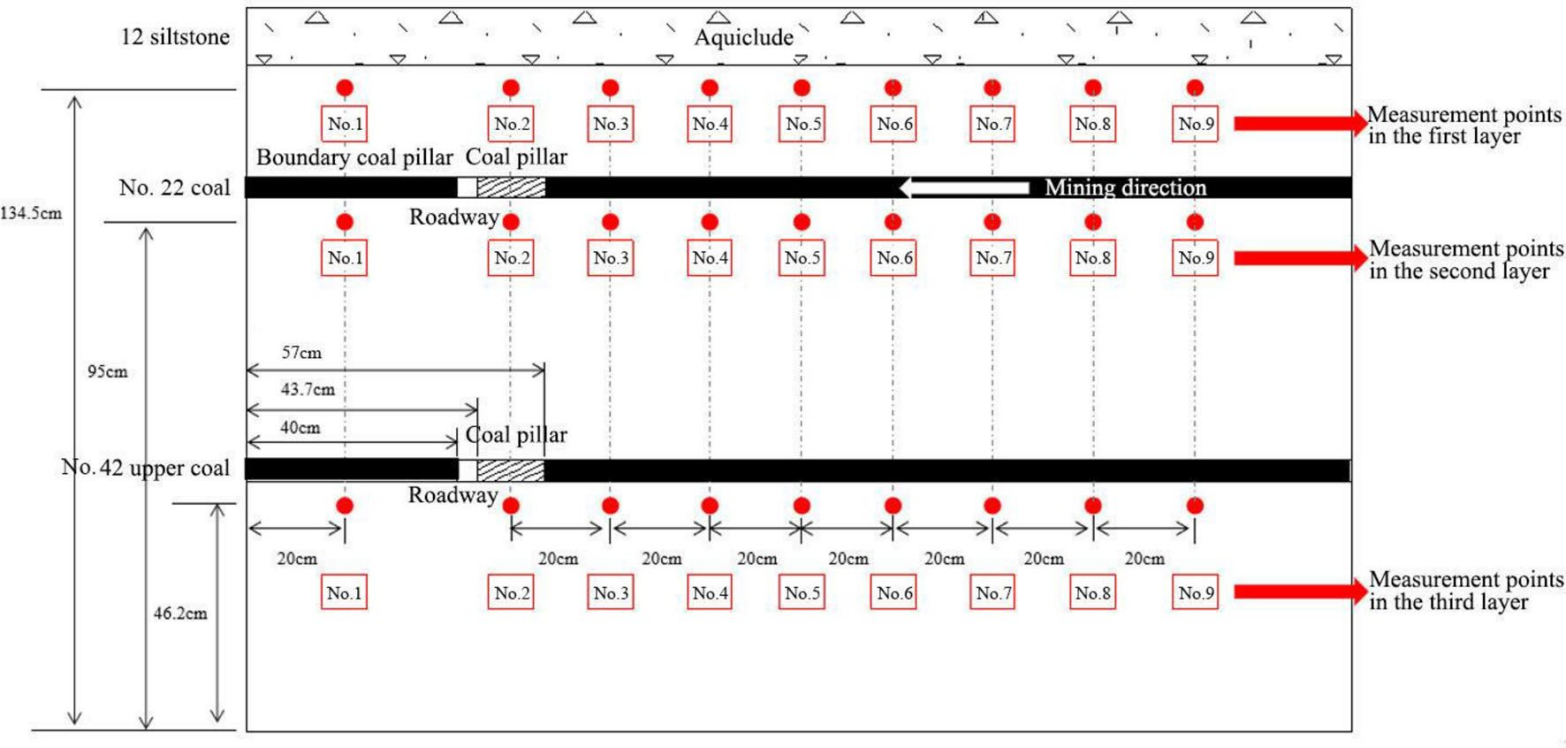
The pressure cell layout illustration.

**Figure 7:**
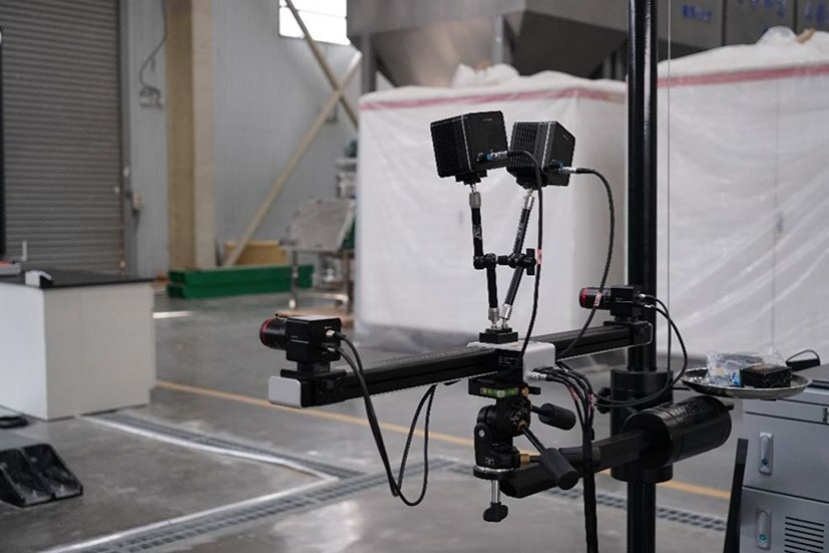
The digital image monitoring equipment (DIC).

The specific procedures to perform the experiment are:

1. The stress measurement lines were arranged on the upper and lower sides of No. 22 coal seam, and the lower side of No. 42 upper coal seam, that is, 134.5 cm, 95 cm and 46.2 cm from the physical model bottom respectively. Pressure cells were arranged each 20 cm on the side line to monitor the vertical and horizontal stress changes in the overlying strata during working face advancement (Fig. 6).
2. The digital image correlation (DIC) technique and the accompanied digital photographic measurement software, PhotoInfor were used to monitor the physical model displacement, stress field and crack evolution (Fig. 7).
3. In the experiment, the surface subsidence, overlying strata movement and coal pillar damage were continuously monitored by the video camera over stepwise excavation.

## Results and analysis

### Overlying strata movement under multiple coal seam mining

#### Overlying strata movement in the early mining stage of No. 22 coal seam

**Figure 8:**
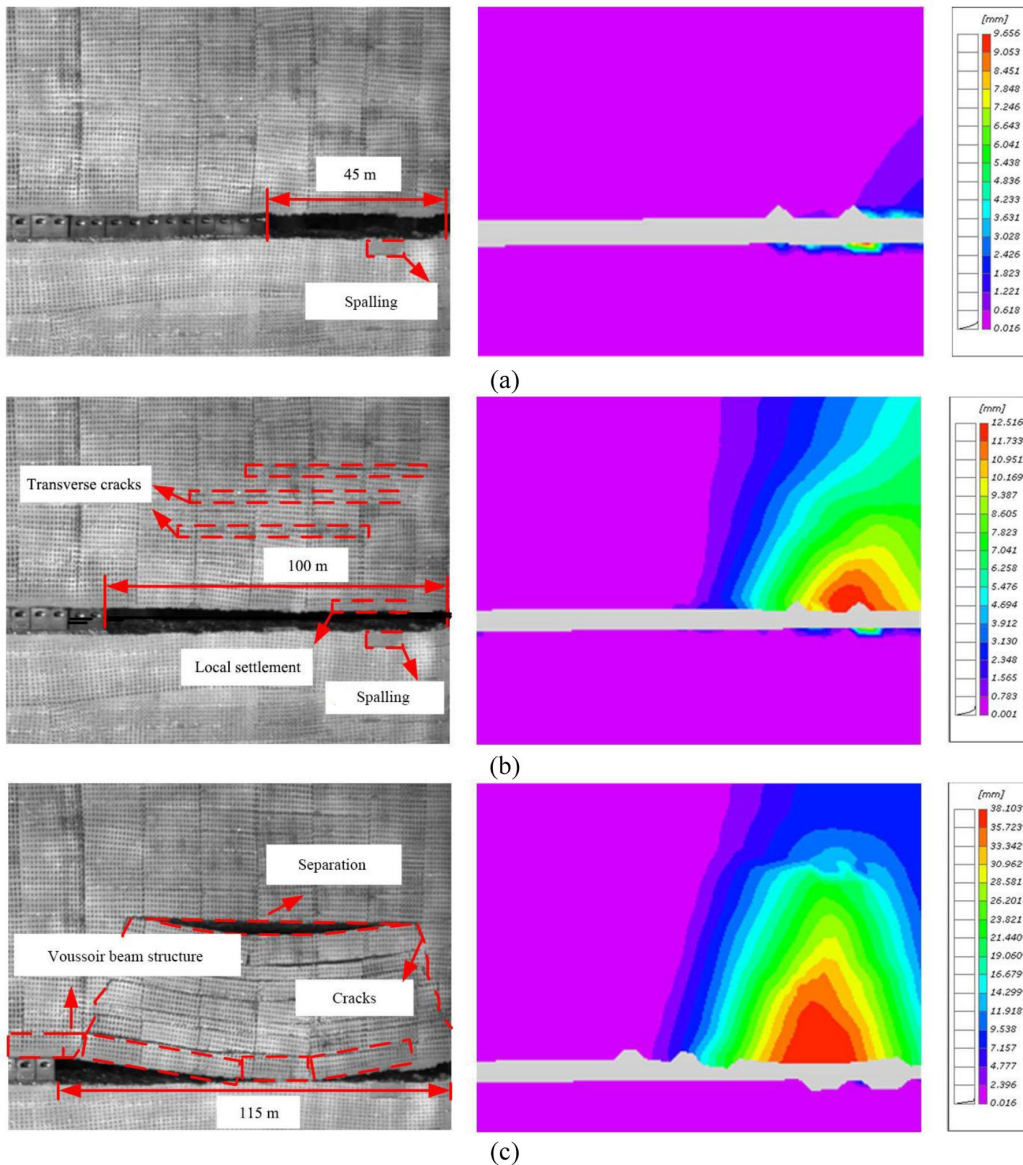
Overlying strata movement in the early mining stage of No. 22 coal seam. (**a**) No. 22 coal seam working face advancement by 45 m; (**b**) No. 22 coal seam working face advancement by 100 m; and (**c**) No. 22 coal seam working face advancement by 115 m.

Following the actual coal mining sequence in Buertai coal mine, the experiment adopted the downward mining method, namely No. 22 coal seam (19.3 mm thickness) was mined first. 1840 kg pressure was loaded to the model top to reproduce an in-situ stress. Then, a roadway was pre-excavated on the left side of the model, and No. 22 coal seam was mined from the right side. When the working face was advanced to 45 m, no remarkable change was observed on the overlying strata structure (Fig. 8a). The left image shows the process of physical simulation experiment, while the right image shows the displacement contours obtained by DIC (Fig. 8a). DIC measurement shows that the roof subsidence was less than 4 mm and the floor displacement was abnormally high due to spalling.

When the working face was advanced by 667 mm (equivalent to 100 m in the field), the stress concentration was formed in roof of 80–220 mm (equivalent to 12–33 m) from the cut, resulting in slight localized subsidence. The average roof subsidence 9 mm with the maximum value of 12 mm (equivalent to 1.8 m in the field). For convenience, in the following, the numbers in the unit of millimeter (mm) and meter represent values in physical modelling and field, respectively. Additionally, new transverse cracks and small separation appeared in the strata at 20–40 m above the roof (Fig. 8b). Meanwhile, the overlying strata also subsided and collapse layer by layer, and the separation were developed between the caved strata and the overlying strata.

As the working face was further advanced, the cracks in roof continued to propagate upwards, and the number of separations gradually increased. When the working face was advanced to 115 m, the main roof subjected to overburden load, broke and rotated, in which the coal wall of the working face was a fulcrum. The large-scale collapse occurred in the strata within 18 m above the goaf, and a significant separation was formed between the main roof and the upper strata. At this time, the caved zone was formed, a voussior beam structure was gradually developed, and the working face underwent the first weighting (Fig. 8c). DIC measurement shows that the maximum subsidence zone was located in the range of 12–33 m away from the cut. As the burial depth decreased, the subsidence of the strata decreased, and the moderate subsidence zone (16–26 mm, equivalent to 2.4–3.9 m in the field) generally resided in the caved zone of the physical model.

#### Overlying strata movement in the middle mining stage of No. 22 coal seam

**Figure 9:**
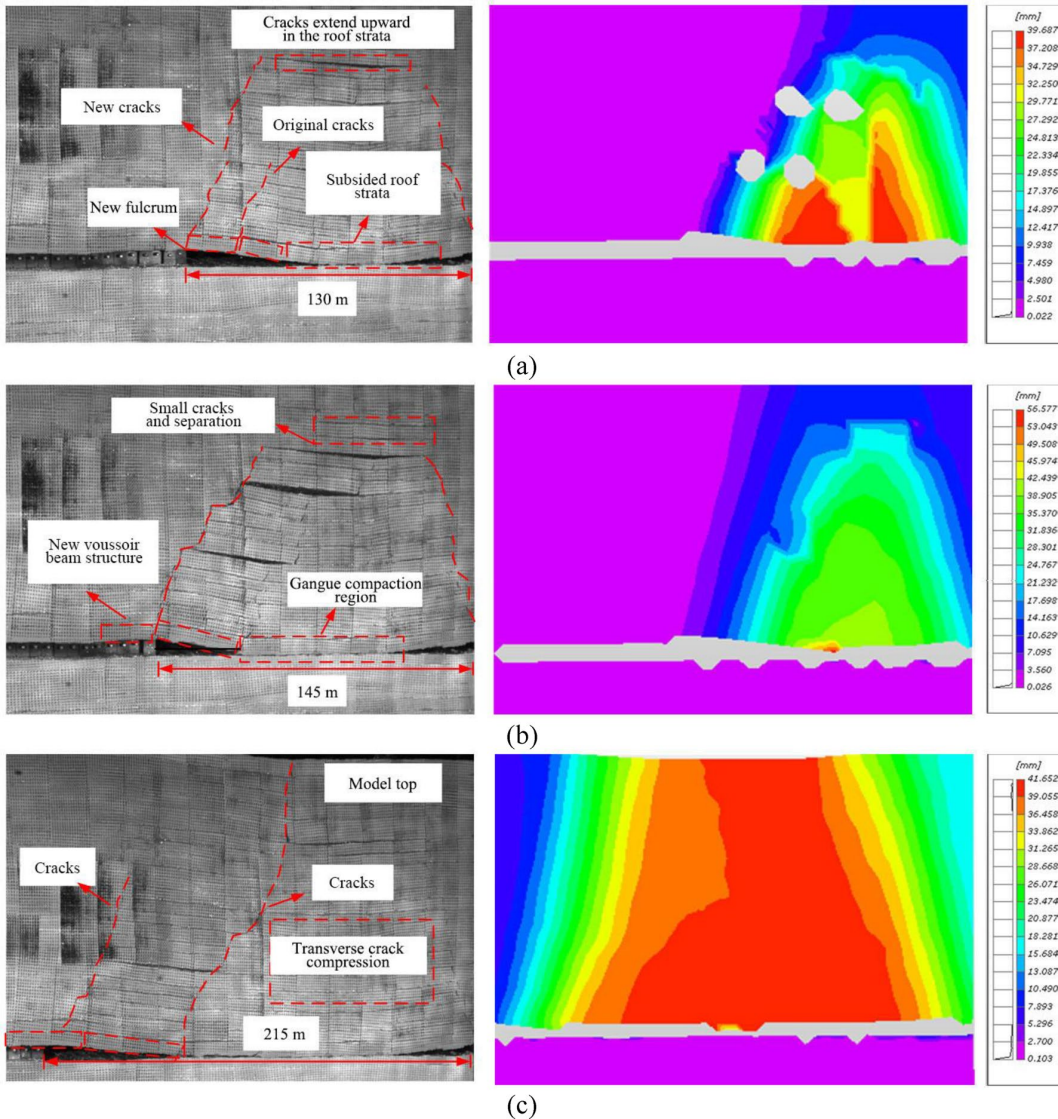
The overlying strata movement in the middle mining stage of No. 22 coal seam working face. (**a**) No. 22 coal seam working face advancement by 130 m; (**b**) No. 22 coal seam working face advancement by 145 m; and (**c**) No. 22 coal seam working face advancement by 215 m.

When the working face was advanced by 130 m (15 m after the first weighting), the main roof rotated slightly with the coal wall as a new fulcrum. A longitudinal crack with an angle of about 20 degrees from the vertical direction appeared at about 6 m from the coal wall, rapidly propagated upward, and coalescence. That is, the angle of draw was initially formed. At the same time, the main roof behind the goaf subsided as a whole and gradually compacted. The separation zone formed by the tensile cracks in the overlying strata was gradually developed upwards, forming a small separation zone at about 82 m above the roof (Fig. 9a).

When the working face was advanced by 145 m (30 m after the first weighting), the separation zone continued to develop upwards and the transverse tensile cracks gradually became narrower. The overlying strata movement tended to be stable at 30–40 m above the working face, and the gangue compaction zone appeared in the goaf. DIC measurement shows that most of the displacement in this compaction zone was gentle except for some surge points. Meanwhile, the main roof was broken and rotated with the coal wall as a fulcrum, forming a new voussoir beam structure and the periodic weighting happed to the working face. With continuous advancement of the working face, the separation formed by the tensile cracks in the overlying strata moved upward, but the width of cracks and separation decreased (Fig. 9b).

When the working face was advanced by 215 m, the significant longitudinal cracks 60 m above the working face continued to propagate upwards and connected the model top (equivalent to the surface in the field). The transverse tensile cracks and separation are gradually closed, and the strata from the roof to the surface experienced integrated subsidence. The maximum subsidence was about 5 m which was close to the mining height. The goaf at 60 m above the working face reached a completely stable state (Fig. 9c). The cracks within 20 m behind the working face gradually developed, expanded and coalesced into new cracks. In the middle and late stages of No. 22 coal seam mining, the overlying strata were in the transition from new crack development to significant tensile crack closure. When the model top subsided to the maximum value, the overlying strata re-entered into a stable state.

#### Overlying strata movement in the late mining stage of No. 22 coal seam

After the model top subsidence occurred, the working face entered into the normal mining stage. In this stage, with the continuous advancement of the working face, new cracks appeared in the overlying strata, and the separation continued to propagate upwards, while the original separations and fractures in the goaf tended to be closed again. The fractures and separations alternately underwent the cycle of “fracture formation-fracture coalescence-fracture closure” over the advancement of the working face. Due to the overlying strata movement behind the working face, a dynamic pressure zone, a gradual compacting zone and a compacted zone were developed.

**Figure 10:**
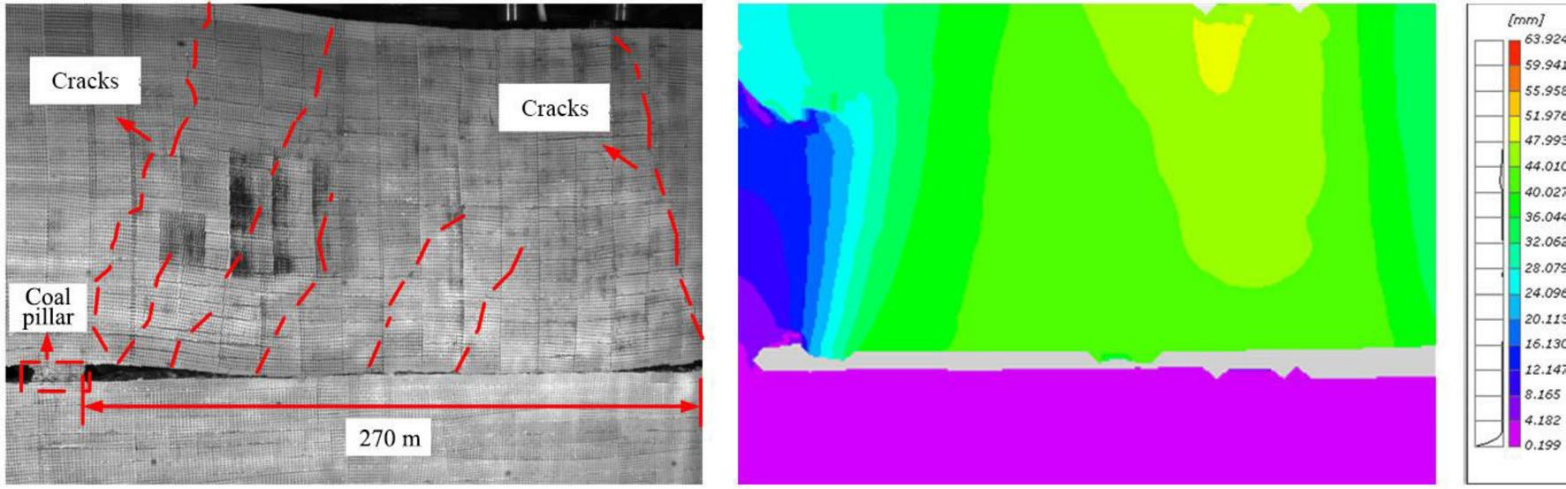
The overlying strata movement in the late mining stage of No. 22 coal seam.

When the working face was advanced by 270 m (i.e., final mining stage), the overlying strata caved at an approximate equal length, and the main longitudinal cracks propagated upwards and connected to the model top (Fig. 10). The overlying strata within 60 m behind the working face was stable, the subsidence was roughly 5 m (field), and the separation and cracks were closed. Over the whole mining of No. 22 coal seam mining, several macroscopic fractures intersected the strata by approximate equal length, causing the periodic weighting of the working face. After termination of No. 22 coal seam, significant damage and fractures did not occur on the coal pillar surfaces.

#### Overlying strata movement in the early mining stage of No. 42 upper coal seam

**Figure 11:**
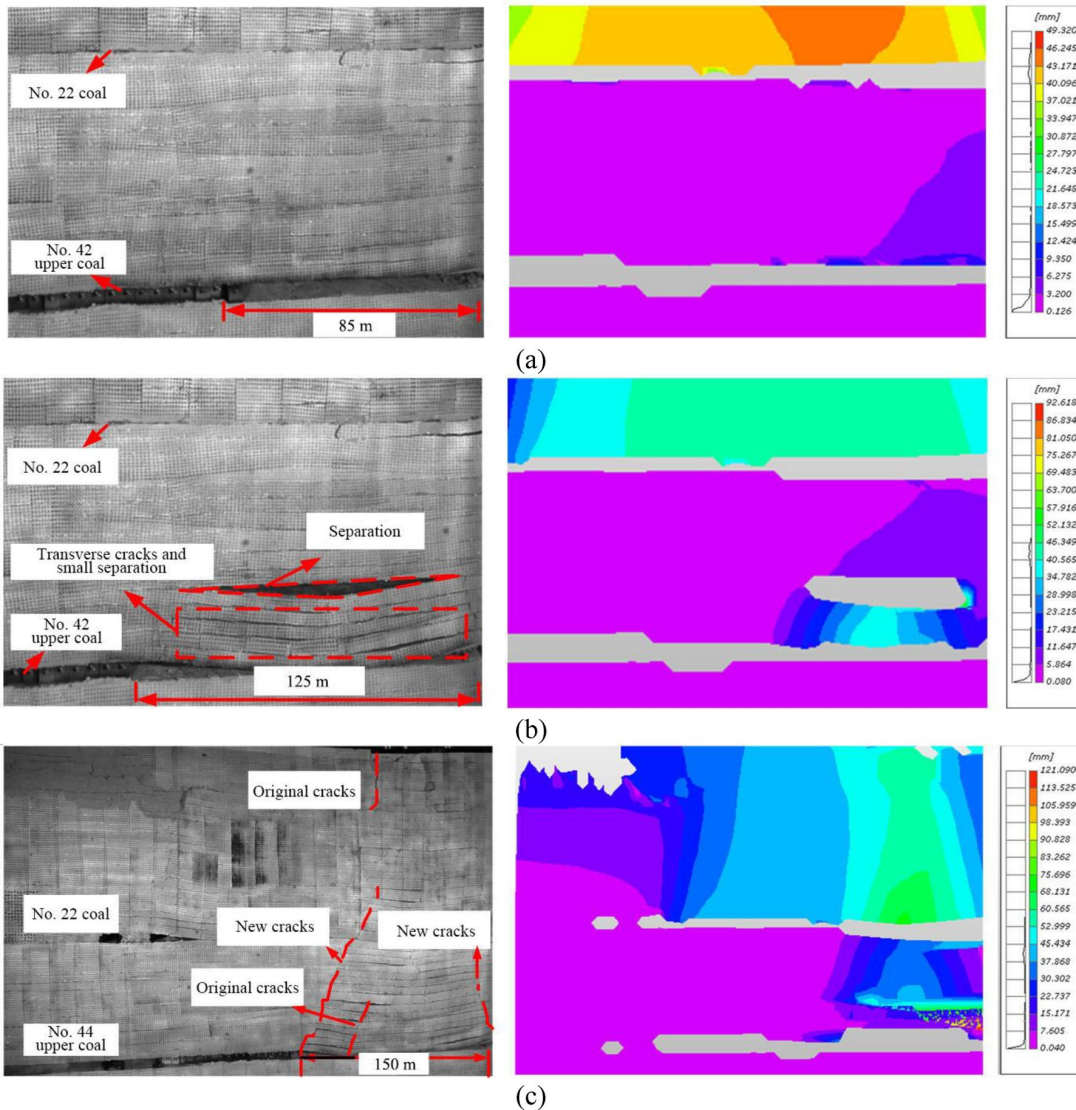
The overlying strata movement in the early mining stage of No. 42 upper coal seam. (**a**) No. 42 upper coal seam working face advancement by 85 m; (**b**) No. 42 upper coal seam working face advancement by 125 m; and (**c**) No. 42 coal seam working face advancement by 150 m.

After No. 22 coal seam mining, No. 42 upper coal seam roadway was excavated, preceding the mining of No. 42 upper coal seam (40.6 mm thickness) from right to left. When the working face was advanced 85 m (Fig. 11a), the structural characteristics of the overlying strata of No. 22 coal seam were basically unchanged, and obvious transverse cracks and separation were not observed. The maximum subsidence of the overlying strata of No. 42 upper coal seam (i.e., inter-coal-seam strata) was less than 0.6 m.

When the working face was advanced by 125 m (Fig. 11b), the inter-coal-seam strata as fixed beams broke and collapsed in the middle where the pressure and moment were maximum, and contacted the gangue in the goaf, causing an excessively suspended roof span and the first weighting of the working face of No. 42 upper coal seam. Transverse cracks and small separation appeared in the inter-coal-seam strata, and significant separation occurred 30 m above the coal seam roof, forming a caved zone. The DIC measurement shows that the maximum subsidence of the inter-coal-seam strata was around 5 m, while that of the overlying strata of No. 22 coal seam experienced unnoticeable change, indicating that the inter-coal-seam strata still had the capacity to support the overburden load. The load supported by No. 42 upper coal seam roof was the sum of the inter-coal-seam strata weight and the gangue weight in the caved zone of No. 22 coal seam.

When the working face of No. 42 upper coal seam was advanced 150 m (Fig. 11c), new cracks occurred at about 25 m in front of the original cracks. The inter-coal-seam strata broke and rotated with the coal wall as a fulcrum, and the working face underwent periodic weighting. The new cracks rapidly propagated upwards to No. 22 coal seam and further to the model top, causing the secondary subsidence of the overlying strata. According to the DIC measurement, the maximum subsidence of interlayer strata is about 6 m, and the overlying strata subsided again by about 3 m. The inter-coal-seam strata lost supporting ability. The overlying strata and the inter-coal-seam strata contacted the gangue and was gradually compacted at about 40 m behind the working face, so the working face entered into the regular mining stage.

#### Overlying strata movement in the late mining stage of No. 42 upper coal seam

**Figure 12:**
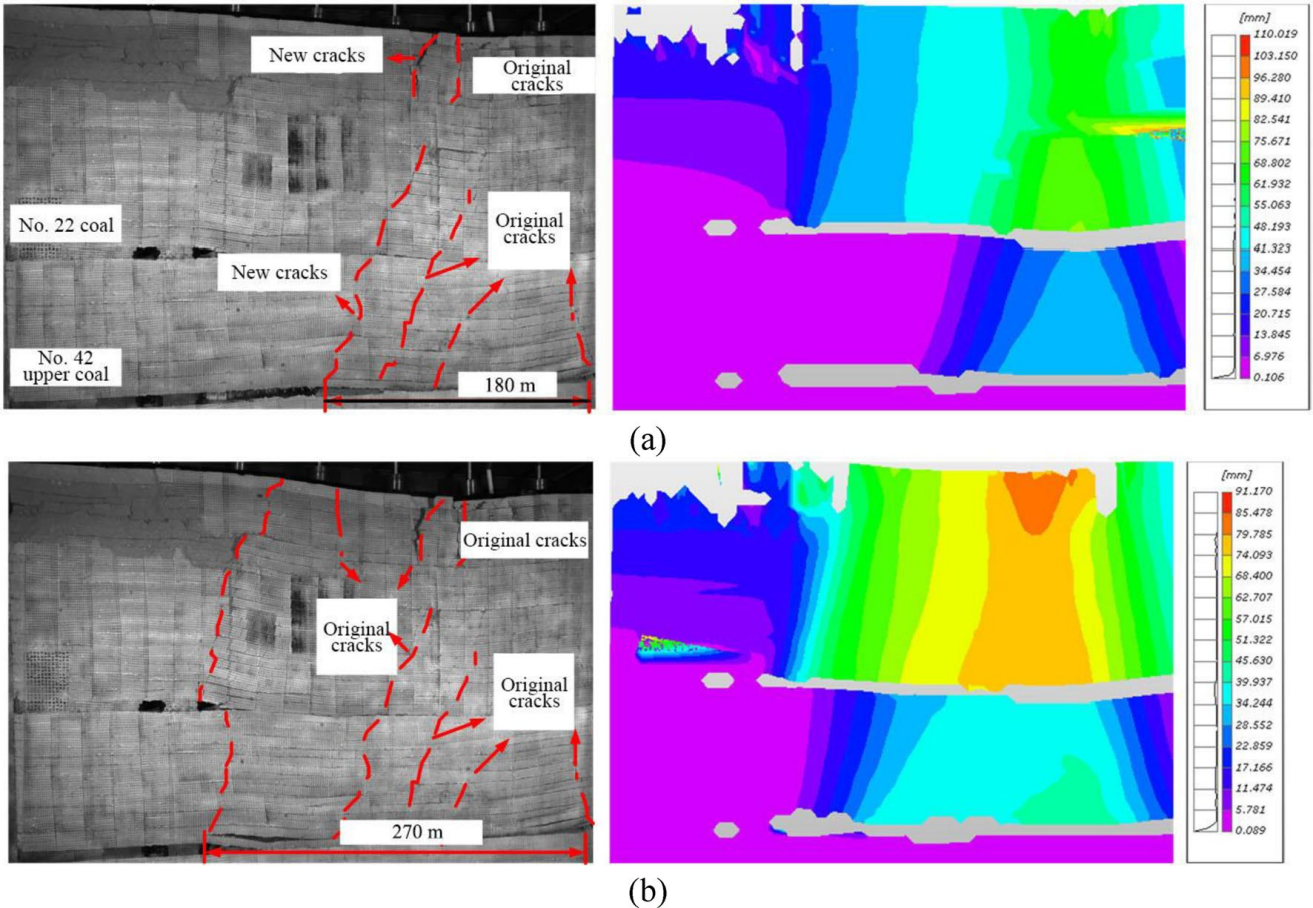
The overlying strata movement in the late stage of No. 42 upper coal seam mining. (**a**) No. 42 upper coal seam working face advancement by180 m; and (**b**) No. 42 upper coal seam working face advancement by 270 m.

When the working face of No. 42 upper coal seam was advanced 180 m (Fig. 12a), the inter-coal-seam strata still broke periodically in a similar span of 25–30 m. The new and original cracks approximately divided the overlying strata into a span of 25–30 m. The inter-coal-seam strata and the overlying strata of No. 22 coal seam were gradually subsiding and compacted, and the working face experienced periodic weighting. According to the DIC measurement, the overlying strata above 40–80 m from the working face continued to subside synchronically, while no significant change was observed on the overlying strata of more than 80 m above the working face and a stable compaction state was arrived. When the working face was advanced 270 m (i.e., termination of No. 42 upper coal seam), due to the high advancing speed, a new crack was formed directly at the coal wall and reached the model top, resulting in the overall subsidence of inter-coal-seam strata and the overlying strata of No. 22 coal seam (Fig. 12b). The maximum subsidence of the inter-coal-seam strata and the overlying strata of No. 22 coal seam were about 5 m and 11.4 m.

During the mining of No. 42 upper coal seam, due to the large thickness of the inter-coal-seam strata, the working face of No. 42 upper coal seam bears less loading and has a marginal impact on the safety of the working face. With the advancement of the working face, the inter-coal-seam strata were deformed, fractured, rotated and collapsed, leading to further increase of the overlying strata subsidence. Since the inter-coal-seam strata was thick, the caved gauge of the inter-coal-seam strata adequately filled the goaf. Although the overlying strata behind the working face still experienced deformation and rotation, a proportion of the overburden load was transferred to the floor. But the distance of the overlying strata in movement to the working face of No. 42 upper coal seam was sufficiently large, thus the impact of the overlying strata movement on the working face pressure was slight.

The digitized recording of the experiment and DIC measurement showed that after mining of No. 22 coal seam and No. 42 upper coal seam, the structural characteristics of two coal pillars changed slightly, and no cracks and separations were induced.

### Stress field evolution of strata under multiple coal seam mining

To monitor the stress field evolution of the overlying strata during the physical modelling experiment, 27 pressure cells were set up in prescribed strata. Among them, two measuring lines with 18 pressure cells were arranged in the roof and floor of No. 22 coal seam, and a measuring line with 9 pressure cells were arranged in the floor of No. 42 upper coal seam. The pressure cell layout is illustrated in Fig. 6.

#### Stress variation of No. 22 coal seam roof

The pressure cells of each measuring line in the coal seam overlying strata are discrete due to different burial positions. Statistical analysis found that most of the data were approximately constant due to the impact of rock movement and pressure cell state. Therefore, representative data were selected for analysis (measurements of damaged and abnormal pressure cells were excluded during data processing).

**Figure 13:**
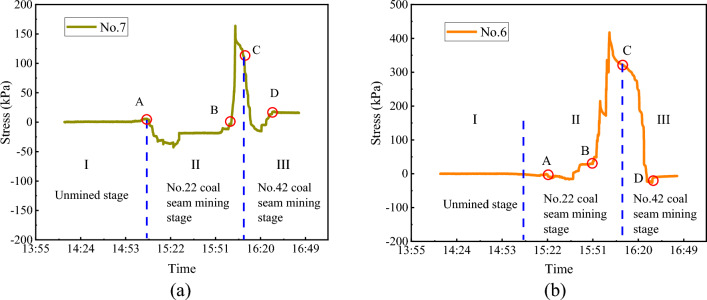
No. 22 coal seam stress evolution. (**a**) Roof; and (**b**) floor.

Figure 13a shows that in the unmined stage, the roof of No. 22 coal seam was only affected by the original strata stress, and the vertical stress was maintained at about 0 kPa. It should be noted that the working principle of the pressure cell resembles the deformation of an elastic body. In the early stage of the physical model construction, the pressure cell was actually subjected to pressing by the upper and lower strata and the pressure cell treated it as the original stress state (with a reading of 0 kPa). When No. 22 coal seam was mined (point A in Fig. 13a), the rock stratum where No. 7 pressure cell was located remained stable in a short period, and the bottom was suspended to form a fixed beam, which reduces the squeezing pressure in the stratum. Thus, the stress recorded by No. 7 pressure cell declined. Since excavation released the squeezing pressure by the strata, the pressure cell reading less than zero was normal.

As the working face of No. 22 coal seam was further advanced, after the roof reached the limited span, it broke and contacted the gangue. The stress in the stratum increased and reached the maximum at 163 kPa (point B in Fig. 13a). In the early mining stage of No. 42 upper coal seam, the inter-coal-seam strata still possessed a certain bearing capacity, and the stress of No. 22 coal seam roof was maintained at a high level. When the inter-coal-seam strata lost its bearing capacity, the roof of No. 22 coal seam underwent rotational instability again; the stress inside the roof commenced to decrease (point C in Fig. 13a), and tended to be stable after compaction in the goaf of No. 42 upper coal seam (point D in Fig. 13a).

#### Stress variation of No. 22 coal seam floor

Figure 13b shows that in the unmined stage, the stress state of No. 22 coal seam floor was only affected by the original strata stress. During No. 22 coal seam mining, No. 6 pressure cell measurement first fluctuated and then decreased. This is because the strata above the coal seam monitored by No. 6 pressure cell was in a suspended state, and there were no gauge/caved strata loaded on the floor of No. 22 coal seam floor, and thus the floor was in a pressure-relief state.

As the working face was further advanced, the roof of No. 22 coal seam collapsed and caved to the goaf. The stress in the floor grew and reached the peak value at 417.75 kPa when the goaf was compacted. When the working face of No. 42 upper coal seam was advanced to a position below No. 6 pressure cell, the inter-coal-seam strata basically has no bearing capacity, and the floor of No. 22 coal seam underwent secondary rotational instability. No. 6 pressure cell measurement fluctuated briefly, then decreased rapidly, and finally stabilized when the compaction zone was formed in the goaf of No. 42 upper coal seam.

#### Stress variation of No. 42 upper coal seam floor

**Figure 14:**
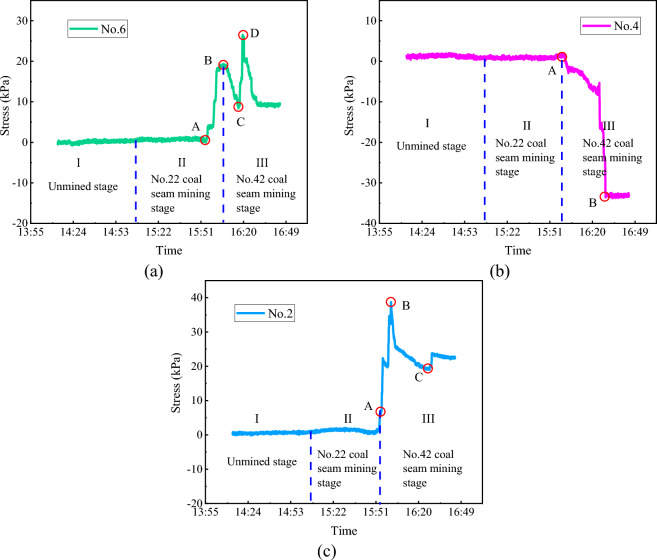
No. 42 coal seam floor stress evolution. (**a**) No. 6 pressure cell; (**b**) No. 4 pressure cell; and (**c**) No. 2 pressure cell.

Figure 14a shows that the stress of No. 42 upper coal seam floor was 0 kPa in the unmined stage and the early mining stage of No. 22 coal seam. With the breakage and collapse of strata above the No. 22 coal seam, the stress of No. 42 upper coal seam floor began to rise (point A in Fig. 14a), and then reached the first peak (point B in Fig. 14a) when No. 22 coal seam roof was compacted.

Before the collapse of No. 42 upper coal seam roof, the zone above the floor was in a pressure-relief state, and the measurement of No. 6 pressure cell decreased and reached the minimum (point C in Fig. 14a) when the hanging roof of No. 42 upper coal seam arrived at the limited span. With the increase of the working face advancement distance, No. 42 upper coal seam roof collapsed and locally contacted locally the gangue in the goaf. The stress of No. 42 upper coal seam floor rose and reached the second peak value at 26.36 kPa (point D in Fig. 14a) under the uncompacted state. As No. 42 upper coal seam continued to advance, the strata above No. 6 pressure cell gradually dropped to the lowest point and then entered into the compaction zone. The stress of No. 42 upper coal seam floor decreased and then became stable over this process.

During excavation, the measurements of most pressure cells in No. 42 upper coal seam floor was similar to that of No. 6 pressure cell, while No. 2 and No. 4 pressure cells displayed different measurements. Figure 14b shows that the measurement of No. 4 pressure cell was 0 kPa in the unmined stage and No. 22 coal seam mining stage. After No. 42 upper coal seam was mined, this data began to decrease and eventually stabilize. This is because the mining of No. 42 upper coal seam released stresses in the strata above No. 4 pressure cell. After the mining of No. 42 upper coal seam was terminated, the floor containing No. 4 pressure cell was located at the end of the working face of No. 42 upper coal seam. Since a voussoir beam structure was formed by the upper strata and the coal pillar, the strata were incompletely collapsed. Consequently, the measurement of No. 4 pressure cell was basically constant without growth.

Figure 14c shows that the measurement of No. 2 pressure cell remained constant over No. 22 coal seam mining, but this measurement grew first and then decreased over No. 42 upper coal seam mining. This is because No. 2 pressure cell was located below the coal pillar, and when the working face of No. 42 upper coal seam reached nearby the coal pillar, a cantilever beam structure with the coal pillar as the fulcrum was formed by the roof. Thus, the measurement of No. 2 pressure cell increased (point A in Fig. 14c) and peaked (point B in Fig. 14c) when the limited span was reached. Later, the strata above No. 42 upper coal seam collapsed, which weakened the supports of the coal pillar and floor as the fulcrum. Consequently, so the measurement of the pressure cell declined and gradually stabilized.

During the mining process of the two coal seams in the physical model, the peak stresses of No. 22 coal seam roof, No. 22 coal seam floor and No. 42 upper coal seam floor were 163 kPa, 417.75 kPa and 26.36 kPa, respectively. The lower coal seam mining increased the subsidence of the upper coal seam roof, whereas the upper coal mining played a noticeable role in pressure relief. Thus, the floor pressure of the lower coal seam was obviously smaller than that of the upper coal seam.

## Conclusions

In this paper, we conducted physical modelling experiments to simulate the excavation process of the No. 42108 working face of Buertai coal mine in China for exploring the overlying strata movement and stress field evolution under multiple coal seam mining. In the physical modelling experiment, the upper-level No. 22 coal seam was excavated first, followed by the excavation of the lower-level No. 42 upper coal seam. Over the successive mining of the two coal seams, DIC (digital image correlation) technique and systematically-arranged pressure cells were employed to examine the overlying strata movement and stress field evolution of roofs and floors.

Our monitoring results showed that the overlying strata of No. 22 coal seam settled gradually over the mining of No. 22 coal seam. When No. 22 coal seam working face was advanced by 45 m, the overlying strata structure basically remained stable; and its overall subsidence was about 5 m as the advancement distance arrived at 270 m. In this process, fractures continuously appeared, propagated and coalesced across the overlying strata. Several remarkable fractures approximately intersected the overlying strata, resulting in periodic weighting of main roof.

In the unmined stage, the overlying strata of No. 22 coal seam and the inter-coal-seam strata (i.e. the overlying strata of No. 42 upper coal seam) were only affected by the initial stress. The stress of the overlying strata of No. 22 coal seam gradually decreased in the early mining stage of the No. 22 coal seam, due to formation of the fixed beam; and it suddenly rose to peak as the strata suspending span grew and finally broke. Then the overlying strata stress gradually decreased and became stable over the mining of No. 42 upper coal seam. The inter-coal-seam strata stress increased significantly in the late mining stage of No. 22 coal seam due to the strata collapse, and peaks when the caved blocks were compacted. Then, the inter-coal-seam stress gradually decreased over mining of No. 42 upper coal seam and reached zero after the collapse of inter-coal-seam strata. The mining of No. 42 upper coal seam on one hand elevated the roof subsidence of No. 22 coal seam; on the other hand, the floor stress was considerably lower than that of No. 22 coal seam due to the pressure-relief caused by preceding mining. This physical modelling study promoted our fundamental understanding on the overlying strata structural characteristics from first weighting to periodic weighting and stress evolutions of roofs and floors over multiple coal seam mining and provided guidance for the working face pressure prediction and mitigation in actual coal mining similar to the layout of Buertai coal mine.

## Data Availability

Thank you very much for the attention to our research. The datasets generated and analysed during the current study are not publicly available due to the company’s confidentiality requirements for the raw data but are available from the corresponding author on reasonable request.

## References

[1] Xu J, Zhu W, Xu J, Wu J, Li Y (2021). High-intensity longwall mining-induced ground subsidence in Shendong coalfield, China. Int. J. Rock Mech. Min. Sci..

[2] Ju J, Xu J (2013). Structural characteristics of key strata and strata behaviour of a fully mechanized longwall face with 7.0 m height chocks. Int. J. Rock Mech. Min. Sci..

[3] Zhao Y, Ling C, Zhang K, Gao Y, Sun B, Wang X (2022). Detection of hidden mining-induced ground fissures via unmanned aerial vehicle infrared system and ground-penetrating radar. Int. J. Rock Mech. Min. Sci..

[4] Ju J, Xu J (2015). Surface stepped subsidence related to top-coal caving longwall mining of extremely thick coal seam under shallow cover. Int. J. Rock Mech. Min. Sci..

[5] Ju J, Xu J, Zhu W (2015). Longwall chock sudden closure incident below coal pillar of adjacent upper mined coal seam under shallow cover in the Shendong coalfield. Int. J. Rock Mech. Min. Sci..

[6] Li Z, Yu S, Zhu W, Feng G, Xu J, Guo Y, Qi T (2020). Dynamic loading induced by the instability of voussoir beam structure during mining below the slope. Int. J. Rock Mech. Min. Sci..

[7] Palchik V (2003). Formation of fractured zones in overburden due to longwall mining. Environ. Geol..

[8] Miao X, Cui X, Xu J (2011). The height of fractured water-conducting zone in undermined rock strata. Eng. Geol..

[9] Le TD, Oh J, Hebblewhite B, Zhang C, Mitra R (2018). A discontinuum modelling approach for investigation of longwall top coal caving mechanisms. Int. J. Rock Mech. Min. Sci..

[10] Arasteh H, Esmaeili K, Saeedi G, Farsangi MAE (2022). Discontinuous modeling of roof strata caving in a mechanized longwall mine in tabas coal mine. Int. J. Geomech..

[11] Wang F, Xu J, Xie J (2019). Effects of arch structure in unconsolidated layers on fracture and failure of overlying strata. Int. J. Rock Mech. Min. Sci..

[12] Lou, J., F. Gao, J. Yang, Y. Ren, J. Li, X. Wang, and L. Yang, Characteristics of evolution of mining-induced stress field in the longwall panel: Insights from physical modeling. *Int. J. Coal Sci. Technol.* 1–18 (2021).

[13] Xu Y, Wu K, Li L, Zhou D, Hu Z (2019). Ground cracks development and characteristics of strata movement under fast excavation: A case study at Bulianta coal mine, China. Bull. Eng. Geol. Environ..

[14] He C, Lu W, Zha W, Wang F (2021). A geomechanical method for predicting the height of a water-flowing fractured zone in a layered overburden of longwall coal mining. Int. J. Rock Mech. Min. Sci..

[15] Wang F, Jiang B, Chen S, Ren M (2019). Surface collapse control under thick unconsolidated layers by backfilling strip mining in coal mines. Int. J. Rock Mech. Min. Sci..

[16] Iwanec AS, Carter J, Hambleton J (2016). Geomechanics of subsidence above single and multi-seam coal mining. J. Rock Mech. Geotech. Eng..

[17] Ren H-F, Cao P, Zhao X-W (2021). Strata movement and fracture evolution characteristics in adjacent seam mining. Arab. J. Geosci..

[18] Li Y, Ren Y, Peng SS, Cheng H, Wang N, Luo J (2021). Measurement of overburden failure zones in close-multiple coal seams mining. Int. J. Min. Sci. Technol..

[19] Zhang D, Qi X, Yin G, Zheng B (2013). Coal and rock fissure evolution and distribution characteristics of multi-seam mining. Int. J. Min. Sci. Technol..

[20] Kuang T, Li Z, Zhu W, Xie J, Ju J, Liu J, Xu J (2019). The impact of key strata movement on ground pressure behaviour in the Datong coalfield. Int. J. Rock Mech. Min. Sci..

[21] Guo H, Yuan L, Shen B, Qu Q, Xue J (2012). Mining-induced strata stress changes, fractures and gas flow dynamics in multi-seam longwall mining. Int. J. Rock Mech. Min. Sci..

[22] Dai S, Han B, Liu S, Li N, Geng F, Hou X (2020). Neural network–based prediction methods for height of water-flowing fractured zone caused by underground coal mining. Arab. J. Geosci..

[23] Yang T, Xu T, Liu H, Tang C, Shi B, Yu Q (2011). Stress–damage–flow coupling model and its application to pressure relief coal bed methane in deep coal seam. Int. J. Coal Geol..

[24] Guo H, Todhunter C, Qu Q, Qin Z (2015). Longwall horizontal gas drainage through goaf pressure control. Int. J. Coal Geol..

[25] Sun Y, Zuo J, Karakus M, Liu L, Zhou H, Yu M (2021). A new theoretical method to predict strata movement and surface subsidence due to inclined coal seam mining. Rock Mech. Rock Eng..

[26] Yang W, Lin B-Q, Qu Y-A, Li Z-W, Zhai C, Jia L-L, Zhao W-Q (2011). Stress evolution with time and space during mining of a coal seam. Int. J. Rock Mech. Min. Sci..

[27] Cheng G, Chen C, Li L, Zhu W, Yang T, Dai F, Ren B (2018). Numerical modelling of strata movement at footwall induced by underground mining. Int. J. Rock Mech. Min. Sci..

[28] Alejano L, Ramirez-Oyanguren P, Taboada J, Rodriguez I (1998). Numerical prediction of subsidence phenomena due to flat coal seam mining. Int. J. Rock Mech. Min. Sci..

[29] Yasitli N, Unver B (2005). 3D numerical modeling of longwall mining with top-coal caving. Int. J. Rock Mech. Min. Sci..

[30] Ju Y, Wang Y, Su C, Zhang D, Ren Z (2019). Numerical analysis of the dynamic evolution of mining-induced stresses and fractures in multilayered rock strata using continuum-based discrete element methods. Int. J. Rock Mech. Min. Sci..

[31] Ren S, Cui F, Zhao S, Cao J, Bai J, Jiang Z, Li Y (2021). Investigation of the height of fractured water-conducting zone: A case study. Geotech. Geol. Eng..

[32] Tan Y, Liu X, Ning J, Lu Y (2017). In situ investigations on failure evolution of overlying strata induced by mining multiple coal seams. Geotech. Test. J..

[33] Zhu W, Xu J, Li Y (2017). Mechanism of the dynamic pressure caused by the instability of upper chamber coal pillars in Shendong coalfield, China. Geosci. J..

[34] Cheng G, Ma T, Tang C, Liu H, Wang S (2017). A zoning model for coal mining-induced strata movement based on microseismic monitoring. Int. J. Rock Mech. Min. Sci..

[35] Hu C, Apel D, Sudak LJ, Liu WV, Chu Z (2020). Physical investigation on the behaviours of voussoir beams. J. Rock Mech. Geotech. Eng..

[36] Ghabraie B, Ren G, Smith J, Holden L (2015). Application of 3D laser scanner, optical transducers and digital image processing techniques in physical modelling of mining-related strata movement. Int. J. Rock Mech. Min. Sci..

[37] Ghabraie B, Ren G, Smith JV (2017). Characterising the multi-seam subsidence due to varying mining configuration, insights from physical modelling. Int. J. Rock Mech. Min. Sci..

[38] Qin Y, Xu N, Zhang Z, Zhang B (2021). Failure process of rock strata due to multi-seam coal mining: Insights from physical modelling. Rock Mech. Rock Eng..

[39] Cheng G, Yang T, Liu H, Wei L, Zhao Y, Liu Y, Qian J (2020). Characteristics of stratum movement induced by downward longwall mining activities in middle-distance multi-seam. Int. J. Rock Mech. Min. Sci..

[40] Huang Q, Du J, Chen J, He Y (2021). Coupling control on pillar stress concentration and surface cracks in shallow multi-seam mining. Int. J. Min. Sci. Technol..

[41] Suchowerska A, Merifield RS, Carter JP (2013). Vertical stress changes in multi-seam mining under supercritical longwall panels. Int. J. Rock Mech. Min. Sci..

